# Analysis of Bacterial Diversity in Fresh Milk from Commercial Dairy Farms in Xinjiang Based on Metagenomic Sequencing

**DOI:** 10.3390/vetsci13070688

**Published:** 2026-07-15

**Authors:** Yingni Sun, Shuyun Xu, Zhijie Luo, Taishan Fan, Xia Zhou

**Affiliations:** 1 Sydney School of Veterinary Science, The University of Sydney, Camperdown, NSW 2135, Australia; 2 College of Animal Science and Technology, Shihezi University, Shihezi 832003, China; 3 Bureau of Agriculture and Rural Affairs of Qapqal County, Ili Kazak Autonomous Prefecture 835300, China

**Keywords:** China, fresh milk, metagenome, microbes, diversity

## Abstract

To investigate the biological characteristics of microbes in fresh milk, this study gathered fresh milk from seven large-scale dairy farms in the southern and northern regions of Xinjiang, analyzing the composition and abundance of bacterial communities in these samples through metagenomic sequencing technology. *Firmicutes*, *Proteobacteria*, and *Actinobacteria* were consistently identified as the dominant phyla across all samples, with stable relative abundance patterns across regions. At the genus level, the genera with the highest relative abundances were *Sporosarcina*, *Streptococcus*, and *Escherichia*, with relative abundances of 2.51–2.58%, 2.23–2.27%, and 1.93–1.97%, respectively. While exploring species richness, it was observed that the XN group had the most OTUs, the DR group had the fewest, and there were significant differences in community structure between the ND group and the other six groups. Further Alpha diversity analysis revealed no significant variation in Chao1 indices across the seven sample groups, highlighting a significant difference in Shannon index for ND samples, and no significant differences in Shannon indices between the CJ, JY, KT, DR, and TR samples. Shifting focus to functional potential, the top three relative abundances in the microbial metagenome KEGG functional library are biological systems, human diseases, and environmental information processing; additionally, within the CAZy (Carbohydrate-Active enZymes) database, the three most abundant categories are glycosyltransferases (GT), glycoside hydrolases (GH), and carbohydrate-binding modules (CBM). By delineating these patterns, this study demonstrates the microbial spectrum characteristics of fresh milk from southern and northern Xinjiang, China, offering a theoretical foundation for enhancing the quality of fresh milk in the area.

## Simple Summary

Fresh milk contains diverse microbiota that can influence milk quality, safety, and processing characteristics. In this study, metagenomic sequencing was used to analyze the microbial composition and functional potential of fresh milk collected from seven large-scale dairy farms in southern and northern Xinjiang, China. The results showed that *Bacillota*, *Pseudomonadota*, and *Actinomycetota* were the dominant bacterial phyla, while *Sporosarcina*, *Streptococcus*, and *Escherichia* were the most abundant genera. Differences in microbial richness and community structure were observed among farms, with the ND group showing the most distinct microbial diversity. Functional analysis revealed that microbial genes were mainly associated with biological systems, human diseases, and environmental information processing. In the CAZy database, glycosyltransferases, glycoside hydrolases, and carbohydrate-binding modules were the most abundant enzyme-related categories. These findings provide new insights into the microbial characteristics of fresh milk in Xinjiang and offer a scientific basis for improving milk quality, safety control, and dairy production management in the region.

## 1. Introduction

Fresh milk is highly nutritious and hosts complex microbiota, the structure of which is affected by numerous factors, with different microbes entering the milk via various pathways. Microbial composition in raw milk is influenced by both the cows’ rearing environment and host-associated endogenous factors, resulting in diverse microbial communities in milk [1,2,3]. The variation in microbiota directly impacts the quality of fresh milk, including its fat and protein content. Different microbiota perform different functions, such as facilitating fermentation, causing spoilage, and affecting animal health and disease development [4].

With the advancement of genetic sequencing technologies, metagenomic sequencing has rapidly developed and is used to study the composition and potential functions of microbiota, playing a significant role in various research and application fields, and paving new paths for microbial diversity studies [5,6,7]. Skeie [8] and others used high-throughput sequencing to understand the microbiota in Norwegian farm milk, while Meng Jia [9] and others investigated the mammary gland microbiomes of Charolais, Simmental, and Red Steppe cattle in Inner Mongolia, China, using high-throughput sequencing techniques. Yuan [10] and others analyzed the relationship between microbiota in fresh milk and quality parameters using high-throughput sequencing. Studies show that there are certain differences in microbiota in fresh milk from different regions and sources. Raats [11], Delbès [12], and others conducted diversity analyses of microbes in fresh milk and found that Firmicutes dominate, while Zeng Xueqin [13] and colleagues discovered that Proteobacteria predominate in the fresh milk microbiota in Yunnan. Additionally, the detection of certain genera such as *Pseudomonas* and *Staphylococcus in* fresh milk is significant for milk quality, bovine mammary gland health, and human health [14].

With the increase in dairy farming, Xinjiang has become an important base for fresh milk production in China, making it crucial to thoroughly research and analyze the microbial composition and characteristics of fresh milk. Therefore, this study aims to characterize the microbial composition and functional potential of fresh milk from Xinjiang using metagenomic sequencing, and to investigate microbial interactions and their ecological implications for mammary health.

## 2. Materials and Methods

### 2.1. Sample Collection

Samples were collected from seven large-scale dairy farms located in southern and northern Xinjiang, China, including DR (Shihezi region), KT (Kuitun region), XN (Aksu region), CJ (Yili region), JY (Korla region), TR (Shawan region), and ND (Kashgar region). A total of 28 bulk tank milk samples were obtained across the seven farms, with four independent replicate samples collected from each farm. All farms maintained approximately 3000 lactating dairy cows under comparable housing and management conditions, with a low mastitis prevalence (<3%). Sampling was conducted during the spring season at routine milking sessions (05:00 and 14:00). For each sampling event, 20 mL of bulk tank milk was randomly collected from the automated milking system. All samples were immediately transported under cold conditions and stored at 4 °C prior to further analysis.

### 2.2. Genomic DNA Extraction

DNA extraction was performed using the CTAB method, followed by an analysis of the purity and integrity with 1% agarose gel electrophoresis (AGE). The DNA concentration was accurately quantified using Qubit, and the sample was then diluted with sterile water in a centrifuge tube to achieve an OD value between 1.8 and 2.0. The samples were sent to Shanghai Zhongke New Life Biotechnology Co., Ltd.Shanghai, China for sequencing.

### 2.3. Library Construction and Sequencing

An amount of 1 μg of genomic DNA from the sample was used to construct a library using the NEBNext^®^ Ultra DNA Library Prep Kit for Illumina. The DNA was randomly fragmented into approximately 350 bp pieces using a Covaris ultrasonicator, followed by end repair, A-tailing, adapter ligation, purification, and PCR amplification to complete the library preparation. The library concentration was initially quantified using Qubit 2.0 and then diluted to 2 ng/μL. The insert size of the library was subsequently analyzed using an Agilent 2100, and the effective concentration of the library was accurately quantified using Q-PCR (with an effective library concentration exceeding 3 nM). After the library passed quality control, different libraries were pooled based on their effective concentrations and the required sequencing output, and then sequenced using Illumina PE150.

### 2.4. Bioinformatics Analysis

The analysis was performed on the New Life Cloud platform of Shanghai Zhongke. The scaftigs sequences of the samples were aligned with the MicroNR database to obtain species annotation information for each gene (Unigene), and the number of different bacterial genera in each sample was counted. Based on species annotations, the α-diversity of the fresh milk microbial community was assessed using Chao1, ACE, Shannon, and Simpson indices. Subsequently, β-diversity was evaluated using PCoA analysis based on Bray–Curtis distance. Differences between groups (and within groups) were analyzed using Anosim based on functional abundance, and the Adonis statistical method was utilized to assess the significance of community structure variations among grouped samples. Differences were also analyzed using *t*-tests and Wilcoxon rank-sum tests. Raw reads were quality filtered using fastp v0.23.2, removing reads with Q-score < 20 or length < 50 bp. Host contamination was removed by alignment to the bovine genome using Bowtie2 v2.4.5. Taxonomic annotation was performed using Kraken2 with the MicroNR database. Functional annotation was conducted using KEGG (2023), eggNOG v5.0, and CAZy databases. Alpha diversity indices were calculated using QIIME2 v2023.9, and statistical analyses were performed in R v4.3.1. Multiple comparisons were corrected using the Benjamini–Hochberg false discovery rate (FDR).

## 3. Results

### 3.1. Quality Control Analysis Results

**Figure 1:**
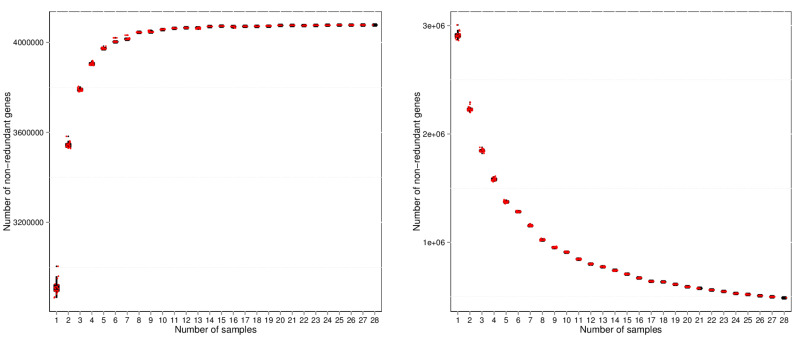
Dilution curve of core and pan gene numbers.

Following shotgun metagenomic sequencing (Illumina PE150 platform) and quality control of 28 fresh milk samples, high-quality reads were obtained for downstream taxonomic and functional analysis, it was found that there are 3,416,799 genes, and effective assembly provided 31,476,546 sequences without N bases, each longer than 500 bp. The total length of the predicted ORFs was 1205.26 Mbp, with an average length of 193.05 bp. The overall base pairs amounted to 6,243,371 with a GC content of 45.13%. Furthermore, 96% of the sequences had a sequencing accuracy of 99% (Q20), demonstrating the high accuracy of the sequencing data. A dilution curve (Figure 1) was plotted with the number of randomly selected samples on the *x*-axis and the count of core and pan genes on the *y*-axis, showing that as the number of samples increased, the numbers of core and pan genes gradually saturated and the curve flattened, indicating that the sequencing data volume is reasonable for further research. This suggests that the genomic diversity is well represented across the samples.

**Figure 2:**
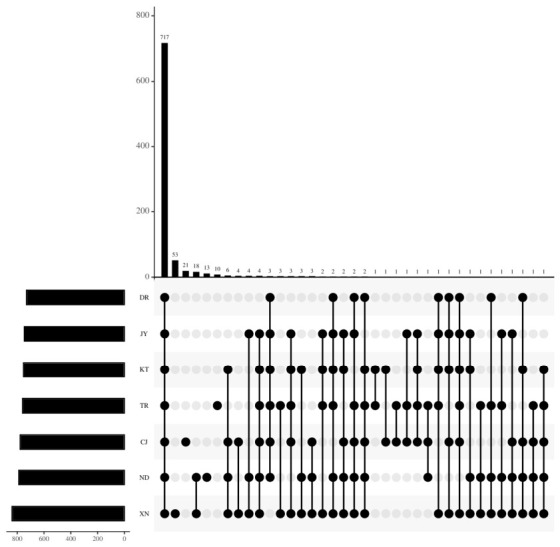
Upset plot of gene sets at the genus level.

The samples contained a total of 3,387,591 microbial genes, with the ND samples having the highest number of unique genes, totaling 12,941. Data visualization (Figure 2) showed that DR, KT, and JY had fewer OTUs. Sample correlation analysis indicates that the parallel sample XN4 within the XN group has a lower correlation with others in the group, and also a low correlation with samples from other groups. The ND group shows low correlation and significant differences with the other six groups. The boxplot of differences in sample gene annotations indicates that the average number of DR gene annotations is lower than that in samples from other regions. This variance highlights potential regional differences in microbial community structures that warrant further investigation.

### 3.2. Results of the Microbial Community Structure and Clustering Analysis in Fresh Milk

#### 3.2.1. Results of Gene Prediction and Abundance Analysis

**Figure 3:**
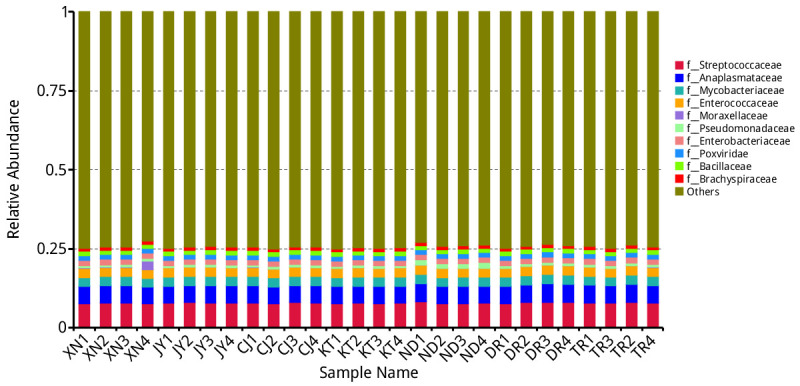

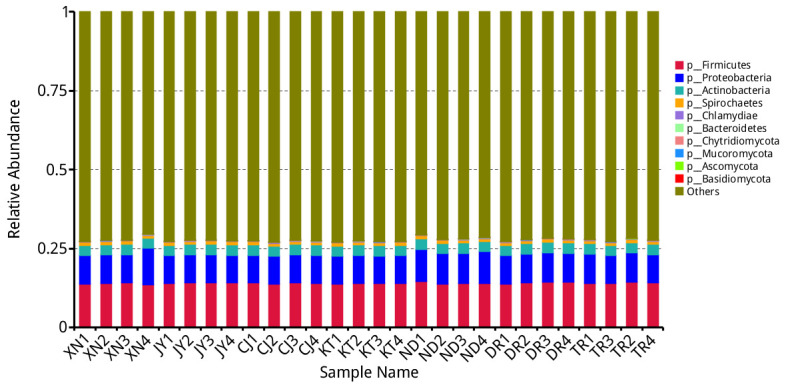

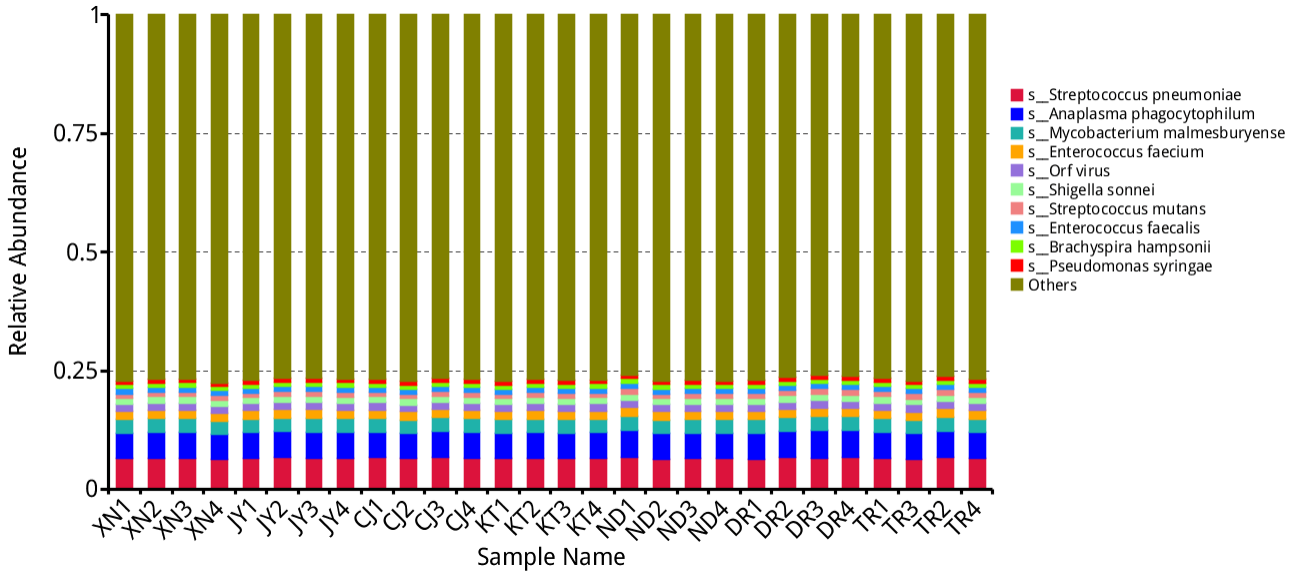
Bar chart of the top ten species’ relative abundance at the phylum, family, and genus levels in fresh milk.

Comparative analysis of microbial community structures revealed substantial differences in the bacterial composition among samples. Microbial sequences obtained from the 28 fresh milk samples were taxonomically assigned to 71 phyla and 891 genera based on high-throughput sequencing data. Among all annotated sequences, 64% remained unclassified at lower taxonomic levels, while bacteria accounted for 34% of the total annotated reads. Species richness was evaluated based on non-redundant gene catalogs and read-based taxonomic annotation, and diversity indices were calculated accordingly. Samples from DR, KT, and XN exhibited higher average OTU counts compared with those from CJ, JY, TR, and ND, with JY showing the lowest OTU richness among all groups. These results suggest regional variation in microbial community diversity and composition across the studied dairy farms. In each group of fresh milk from the seven regions, phyla, families, and genera were identified (ranked top ten in abundance), as shown in Figure 3. Across all samples, the major phyla were *Firmicutes*, *Proteobacteria*, and *Actinobacteria*, constituting 45%, 33%, and 13%, respectively, and representing over 70% of the total bacterial. While there are shared phyla among different fresh milk microbiota, their similarities vary; the relative abundance of Proteobacteria in ND samples (28%) is notably higher than in others, and the OTU counts are consistent within groups across samples. In the Firmicutes phylum, 92% belong to the class Bacilli, of which 88% are of the order *Lactobacillales*. Within the *Proteobacteria*, 62% belong to *Alphaproteobacteria* and 29% to *Gammaproteobacteria*, with significant variance in the distribution of *Pseudomonadales* among the samples. For ND1, ND2, and ND3, *Pseudomonadales* account for 49%; in ND4, 55%; and in other samples, this group represents 32%, which is lower than in the ND samples. The top three families are *Mycoplasmataceae* (3%), *Streptococcaceae* (2%), and *Enterococcaceae* (2%). The three leading genera are *Streptococcus*, *Monascus*, and another unspecified genus, reflecting a diverse microbial environment.

#### 3.2.2. Microbial Community Diversity Based on Alpha and Beta Analysis

**Figure 4:**
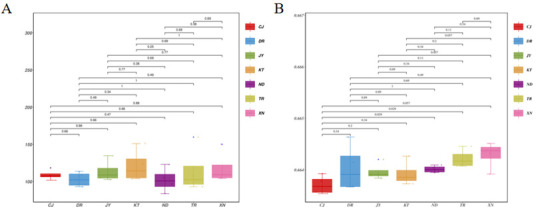
Analysis of microbial alpha diversity in fresh milk: (**A**): The Chao1 index evaluates the richness of the microbial community in fresh milk, (**B**): The Simpson index evaluates the evenness and community coverage of bacteria in fresh milk.

Alpha diversity analysis includes indices such as Chao1, ACE, Shannon, and Simpson, with Chao1 and ACE indices reflecting the richness and number of species in the samples. According to the Chao1 index, there are no significant differences in species richness between the samples from CJ, JY, KT, XN, DR, TR, and ND (*p* > 0.05). The Simpson and Shannon indices primarily reflect the evenness and diversity of bacterial communities. The Shannon indices reveal significant differences between ND, XN, and other regions including CJ, JY, KT, DR, TR (*p* < 0.05), with lower community diversity in ND and XN areas compared to others. Differences among CJ, JY, KT, DR, TR samples are not significant (*p* > 0.05), suggesting similar species diversity and microbiota across these dairy farms (Figure 4A). Comparing regional samples, significant differences exist in Simpson indices between CJ and ND, CJ and TR (*p* < 0.05), with no significant differences in other areas (*p* > 0.05). All samples have Simpson indices between 0.663 and 0.665, with CJ having the lowest average, indicating lower evenness and possibly higher species dominance in CJ region samples (Figure 4B).

**Figure 5:**
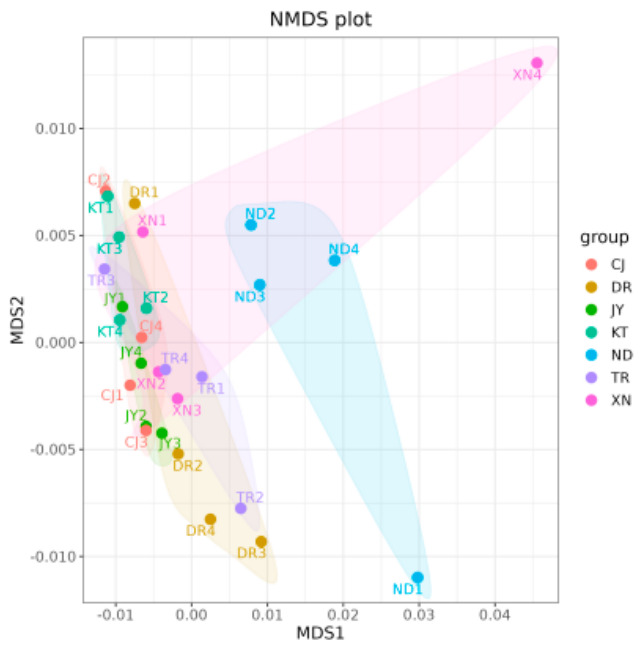
Beta diversity assessment based on Bray–Curtis similarity, NMDS results at the phylum.

**Figure 6:**
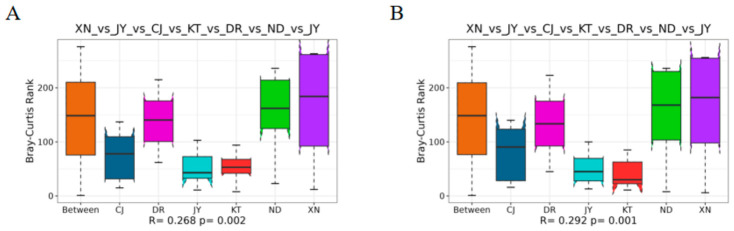
Anosim similarity statistical analysis results based on family level across different groups: (**A**) Analysis results at the phylum level; (**B**) Analysis results at the genus level.

**Figure 7:**
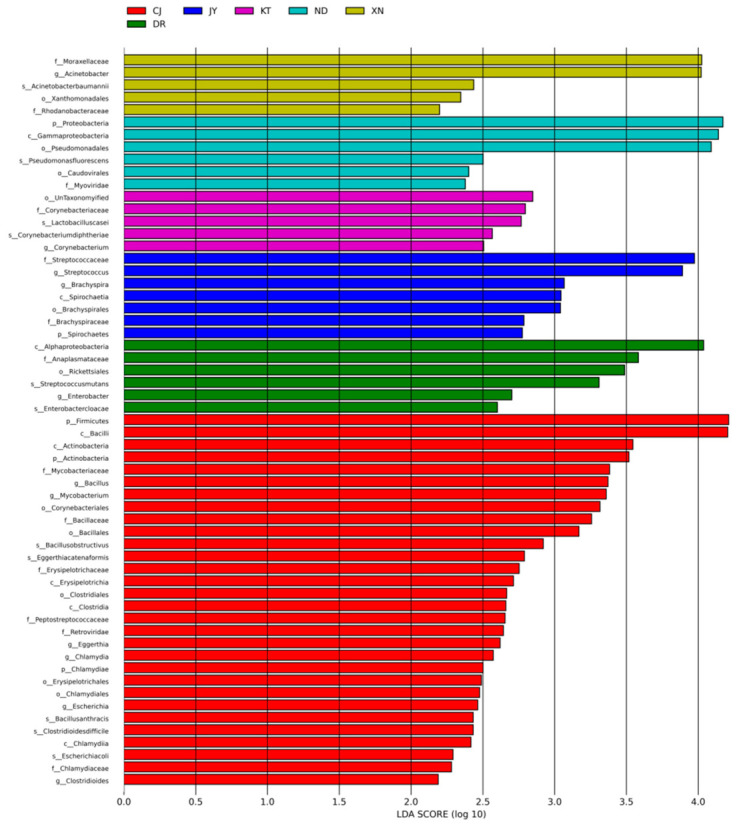
Significant analysis of bacterial Lefse differences between groups.

Beta diversity analysis revealed significant differences in the microbial composition of raw milk at the phylum level (R^2^ = 0.433, *p* = 0.02). ND site samples were more dispersed, and within XN, XN4 had lower similarity with other intra-group samples (Figure 5). At the genus level, significant differences were observed between XN4 and ND1, and samples from other sites (*p* = 0.001). When grouped by sampling region, the distribution within the XN and ND groups in Southern Xinjiang was dispersed, with significant differences, whereas samples from Northern Xinjiang were more clustered. Anosim analysis (Figure 6) showed that the inter-group differences were greater at the family level (R = 0.292) than at the phylum level (R = 0.268). In a significant inter-group bacterial Lefse analysis (Figure 7), notable findings included *Flavobacteriales* and *Acinetobacter* in XN samples, *Pseudomonadales* in ND samples, *Bacillaceae* and *Lactobacillaceae* in KT samples; *Streptococcales* and *Spirochaetes* in JY samples; *Rickettsiales* and *Enterobacteriaceae* in DR samples; and *Actinomycetales*, *Bacillales*, *Bacillus*, *Erysipelothrix*, *Nitrosomonadaceae*, and Chlamydia in CJ samples, all showing significant differences.

The KEGG PATHWAY Database reveals that organismal systems have the highest relative abundance, with sensory systems being the most enriched functional category, followed by the immune system. The core bio-metabolic pathways rank second in relative abundance for human diseases, third for environmental information processing systems, and fourth for metabolism, with amino acid and carbohydrate metabolism having the highest abundance, and cellular processes ranking fifth. The relative abundances of the last three metabolic pathways are closely matched at 15%, 14%, and 11%, respectively, with XN and ND areas showing higher relative abundances within the metabolism group compared to other regions.

**Figure 8:**
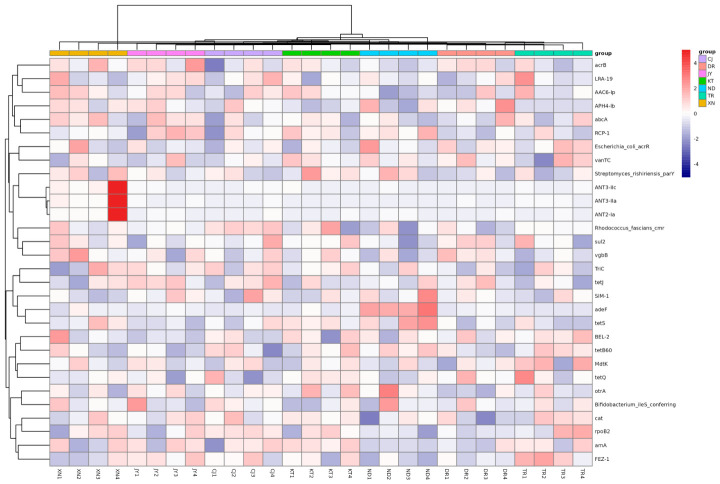
Heatmap clustering of the top 30 ARO resistance genes.

From metagenomic sequencing, 2828 unique CAZy (Carbohydrate-Active enZymes) entries were identified, where glycosyltransferases (GT) showed the highest relative abundance, polysaccharide lyases (PL) the lowest, followed by glycoside hydrolases (GH) and carbohydrate-binding modules (CBM). Within the functional subcategories, GH23 has the highest abundance in the GH family; CBM50, CBM14, and CBM13 are the most abundant in the CBM family. GT4, GT2, GT51, GT27, and GT31 are highly abundant in the GT family, and CE4 is the most abundant in the CE family. GH23, GT4, GT2, GT51, and CE4 were not detected in samples from the JY, CJ, KT, and DR regions. GT4 and CE4 were absent in TR samples. This distribution indicates a regional variability in enzyme presence, which could reflect differences in dietary inputs or microbial community structures (Figure 8).

Analysis of resistance genes (ARO) shows that the ARG with the highest relative abundance in the top 20 is *E. coli*-acrR, which is an Escherichia coli multidrug resistance efflux pump inhibitor gene. This is followed by aminoglycoside resistance genes, including ANT3-IIa, ANT3-IIcA, ANT2-Ia; the carbapenemase resistance gene *SIM-1*; and the tetracycline resistance gene *otrA* (Figure 8). This array of resistance genes highlights the significant challenges in managing antibiotic resistance in microbial populations.

## 4. Discussion

Microbiota in various habitats such as soil, intestines, and food are complex, consisting of phyla like *Firmicutes*, *Proteobacteria*, *Actinobacteria*, and *Bacteroidetes*, influenced by numerous factors. Different species perform diverse functions, and their abundance and compositional changes are closely related to animal health and disease occurrence [15,16,17]. Using metagenomic sequencing, this study analyzed the microbial community diversity in raw milk from large-scale dairy farms across southern and northern Xinjiang, China. The findings revealed that the predominant microbial phyla in raw milk are *Firmicutes*, *Proteobacteria*, and *Actinobacteria*, consistent with the findings by Zhang Ruiyang [18] and others on microbial diversity in northern Xinjiang’s raw milk. At the phylum level, three dominant bacterial phyla with relative abundance greater than 1%: *Firmicutes*, *Proteobacteria* and *Actinobacteria*; *Bacteroidetes* are less than 1%, ranging from 0.38% to 0.4%. Eight microbial families have a relative abundance over 1%: *Mycoplasmataceae*, *Streptococcaceae*, *Enterococcaceae*, *Bacillaceae*, rod-shaped bacteria (specific family name if applicable), *Pseudomonadaceae*, *Enterobacteriaceae*, and *Spiroplasmataceae*. Among these, *Pseudomonadaceae* are particularly abundant in ND samples, exceeding 1%, with other groups having abundances between 0.78% and 0.80%. Bingyao Du [19] identified 116 strains of *Pseudomonas* in cow’s milk from different seasons collected across Inner Mongolia, Heilongjiang, Gansu, Henan, Anhui, Jiangsu, Chongqing, and Hunan, including *Pseudomonas fluorescens*, *Pseudomonas veronii*, and *Pseudomonas psychrophila*. *Pseudomonas*, thrives at low temperatures and secretes extracellular enzymes such as lipases and proteases that can degrade nutritional components of milk. The majority of the *Proteobacteria* are Gram-negative and are divided into five classes: α, β, δ, ε, and γ, each including numerous pathogenic species that can significantly impact the microbial community ecology and the hygiene quality of fresh milk. A farm in the northwest of England experienced an outbreak of 69 cases of campylobacteriosis due to contamination of milk by *Campylobacter* (*ε-Proteobacteria*). From bulk tank milk in Italy, 35.6% of 149 isolated strains of Escherichia coli were identified as pathogenic [20,21]. At the species level, five microbes with a relative abundance greater than 1% include *Tropheryma whipplei*, *Streptococcus*, *Mycobacterium marinum*, *Bacillus obstructus*, and *Enterococcus faecium*. Within the *Firmicutes* phylum, there are both beneficial and pathogenic bacteria such as *Lactobacillus*, *Enterococcus*, *Staphylococcus epidermidis*, *Streptococcus*, *Clostridium*, and *Bacillus*, predominantly composed of Gram-positive bacteria. *Actinobacteria* play a crucial role in the decomposition of organic matter and can produce numerous bioactive metabolites and derivative antibiotics, with over 150 known genera, including Bifidobacterium, an important physiological group in the gut [22,23,24]. Additionally, *Bacteroides* are involved in many vital metabolic activities in the human body, but their migration out of the intestines can lead to various infections and abscesses. Lactic acid bacteria-associated taxa (mainly belonging to the order *Lactobacillales*) were present at low relative abundance within Bacilli-classified sequences in raw milk samples (~4.5%), as estimated from sequencing-based relative abundance profiles, with lactic acid bacteria types having a relative abundance of 0.24%. Lactic acid bacteria like Lactobacillus casei are used as probiotics in the food industry. The types and quantities of bacteria affect the quality of raw milk. For instance, Cremonesi [25] and others, by comparing the microbial diversity in healthy and diseased cow’s milk, found that diseases might lead to a decrease in microbial diversity; similarly, Falentin [26] and others observed significant changes in the microbiota of raw milk due to mastitis, even when the cows were not in an active infection phase. Studies indicate a possible endogenous intestinal-mammary pathway within animals, allowing specific bacteria, like those from the *Enterobacteriaceae* family, to move to the mammary glands [27], which may result in decreased milk quality. The research also reveals that eukaryotes, archaea, and viruses constitute secondary elements of the fresh milk microecosystem. Among fungi, *Ascomycota* (0.017% to 0.021%) and *Basidiomycota* (0.012% to 0.014%) have higher relative abundances, Archaea have a lower relative abundance, and there are 58 types of bacteriophages, with the highest abundance found in the XN sample. Endogenous bacteriophages play a crucial role in host health and disease by clearing bacteria and regulating the human ecosystem [28].

The analysis by Zhengxin Ma [29] and others found significant differences in α and β diversity between two dairy farms in Florida, as well as in the bacterial distribution of fresh milk from different farms. In this research, which involved samples from Southern Xinjiang (ND, XN, TR) and Northern Xinjiang (CJ, DR, JY, KT), the Chao1 index indicated no significant differences in species richness between the regions in α diversity analysis (*p* > 0.05). This was consistent across both regions. According to the Shannon index, however, there is a significant difference (*p* < 0.05) between the ND and XN samples from Southern Xinjiang compared to those from Northern Xinjiang, where ND and XN regions show considerably lower community diversity than other areas. Conversely, farms in Northern Xinjiang exhibit similar species diversity and microbiota. Among the regional samples, the CJ group had the lowest average Simpson index, indicating higher community diversity.

Beta diversity analysis revealed significant differences in the microbial composition of fresh milk at the phylum level (R^2^ = 0.433, *p* = 0.02). In Southern Xinjiang, the ND group samples were more dispersed, with XN4 in the XN group showing low similarity to other samples in the group, whereas samples from Northern Xinjiang were more clustered. In the KEGG PATHWAY Database, the highest relative abundances are found in biological systems, primarily including sensory, immune, and endocrine systems. Human diseases, primarily cancer and infectious diseases, are the second most prevalent category. These KEGG categories represent functional annotation of microbial genes rather than active disease processes in milk samples. The third category is environmental information processing systems, focused primarily on signal transduction and environmental information processing. The metabolism groups rank fourth in relative abundance, particularly amino acid and carbohydrate metabolism, which are the most abundant. Research indicates that carbohydrate-active enzymes (CAZy) are strong predictors of changes in animal microbiota, associated with catalyzing chemical reactions in metabolism, regulating bio-metabolism, and linking specific microbial lineages with particular carbohydrates [30,31].

This study identified 2828 distinct CAZys. Among the six annotated enzyme classes, GT, closely related to glycosidic bond formation, had the highest relative abundance, followed by GH associated with hydrolysis and rearrangement of glycosidic bonds. CBM ranked third, and PL had the lowest relative abundance. These findings underscore the diverse functional capabilities of CAZys in microbiota.

The resistance genes with the highest relative abundance are aminoglycoside resistance genes. Lee Kyungwon [32] and others discovered the *SIM-1* gene in Acinetobacter baumannii isolates, which exhibit a multidrug-resistant phenotype. This gene is notable for its strong association with resistance to multiple antibiotics. The study also found a high abundance of the multidrug resistance gene *MdtK*. Subsequently, research by Bay, Denice C, and colleagues has shown that the transporter proteins encoded by *MdtK* can improve microbial biofilm formation and enhance antimicrobial resistance. This mechanism is crucial as it contributes significantly to the persistence and virulence of bacterial infections.

## 5. Conclusions

This study used metagenomic sequencing to analyze the microbial diversity and functional characteristics of fresh milk collected from seven large-scale dairy farms in northern and southern Xinjiang, China. Results showed that the dominant bacterial phyla were *Firmicutes*, *Proteobacteria*, and *Actinobacteria* across all samples. At the genus level, *Sporosarcina*, *Streptococcus*, and *Escherichia* were the most abundant. Significant differences in microbial community composition and diversity were observed between regions and farms, indicating that geographic location and farm environment can influence milk microbiota. These findings provide insights into the microbial ecology of raw milk and may support quality and safety control strategies implementation.

## Data Availability

The original contributions presented in this study are included in the article. Further inquiries can be directed to the corresponding author(s).
